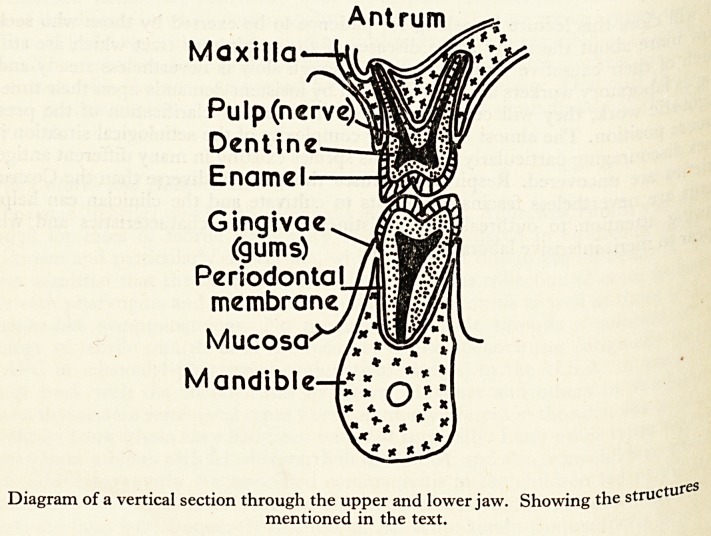# Pain Related to the Teeth and Jaws

**Published:** 1958-04

**Authors:** A. I. Darling

**Affiliations:** Professor of Dental Surgery, University of Bristol


### PAIN RELATED TO THE TEETH AND JAWS

BY

A. I. DARLING

*Professor of Dental Surgery, University of Bristol*

Pain in and around the jaws is most commonly caused by pathological conditions related to the teeth and their supporting structures (*see* diagram) but it can also be caused by disease of other structures in these regions. It is not intended to discuss here such conditions as trigeminal neuralgia, which are well known to the medical practitioner, but rather to consider those local conditions which commonly cause confusion and difficulty in diagnosis.

Diagram of a vertical section through the upper and lower jaw. Showing the structures mentioned in the text.

The precise diagnosis of "dental" pain is often a matter of considerable difficulty. Its localization by the patient is often very poor, making it difficult to demonstrate which tooth, if any, is responsible. It is quite common for the patient to point confidently to a particular tooth as the site of the pain when the teeth themselves are not at fault.

The causes of dental pain are most commonly dental caries and its sequelae, pulpitis, periodontitis and alveolar abscess. Next in frequency come the diseases of the soft tissues, notably gingivitis and trauma from ill-fitting artificial dentures. Another group arises from pathology within the jaws such as buried roots or cysts which have become infected. Finally, and much less frequently, there are the diseases of bone such as osteomyelitis, which affects the lower jaw almost exclusively in adults, and the diseases of the antrum which frequently cause pain related to the maxillary teeth. There are many others, but these are the common causes. Pain can also be referred to the teeth from rather more remote areas of which the most common is the tempero-mandibular joint, while the reference of pain from one jaw to the opposing jaw is quite commonplace.

### DENTAL CARIES AND ITS SEQUELAE

Dental caries, the commonest cause of pain is found in areas of stagnation, and the usual sites are the pits and fissures in the biting surfaces of the posterior teeth, the contact points between neighbouring teeth, and in later life, as the gums begin to recede, the necks of the teeth just above the gums. In these areas the stagnation of carbohydrates, particularly the refined forms such as sugar and sweets, results in their fermentation by bacteria with the production of acid. The acid attacks the enamel, causing decalcification, and burrows through the enamel from a comparatively small point of entry, undermining the enamel surface and often reaching the dentine before any cavity is formed. At this stage the damage is hardly visible by means of X-rays and is certainly not recognizable by clinical examination. Pain usually commences soon after the dentine is involved while the cavity is still very small, and the patient may notice slight pain on eating sweet or salt foods. The precise mechanism of this pain is not known, but it is unlikely that the pulp (i.e. "nerve") would be seriously involved at this stage. As the condition progresses, more dentine is involved and the caries approaches the pulp. The response to sweet foods becomes more severe until eventually the tooth becomes sensitive to thermal changes. This is an indication of early pulpitis, but even so this disease of the pulp may resolve with appropriate treatment. If the condition progresses further, the classical result will be the development of a severe pulpitis with lancinating pain of mounting severity, culminating in an excruciating pain lasting for half to one hour, after which there is a sudden relief. This relief is usually explained on the basis of strangulation of the pulp at the small foramen at the apex of the root where the venules become compressed by the engorged arterioles. At the same time, organisms invade the pulp, and the inflammatory process extends beyond the apical foramen into the periodontal membrane between the root and the bone. This is a periodontitis which in fact is a form of periostitis. The tooth is now tender to sudden pressure from biting or percussion, and as the inflammation progresses the pain becomes more continuous and more severe. It develops a throbbing character which is accentuated on lowering the head or lying down, and is most likely to become acute during sleep. At this stage, organisms have begun the formation of an alveolar abscess and soon the patient may have all the signs of pus under pressure. The tooth is raised in its socket and is knocked by every attempt at mastication with consequent pain. Eventually the pus will burrow through to the surface of the bone with relief of tension and of pain. Usually this pus presents as a "gumboil" within the mouth and over the root of the offending tooth; however, it may present at some distance from the tooth concerned and at times on the face.

This is the classic story as it occurs in the adult. Particularly in children and not uncommonoy in adults, the condition may subside into a chronic phase as a chronic pulpitis or periodontitis. In these circumstances the symptoms regress and the pain produced is often of a dull intermittent character which is difficult to diagnose. In such cases, acute exacerbation is not uncommon.

### GINGIVITIS

Gingivitis, or inflammation of the gums, is fairly easy to recognize. Originally it is usually related to bad oral hygiene with excessive deposits of dental calculus (tartar) on teeth where they meet the gums. These cause a low grade chronic inflammation with swelling of the gum margins so that the gums project beyond the surface of the teeth and extend upwards on to the tooth surface. As they project beyond the tooth surface, they become subject to constant trauma from food in chewing and as they now overlap the tooth surface they create a fine space or pocket between themselves and the teeth in which food and calculus can stagnate, causing further irritation and inflammation. This may remain chronic for many years, but the irritation within the pocket causes ulceration at its base, deepening the pocket and producing an inflammation in the periodontal membrane which leads to its gradual destruction. This is *periodontitis simplex*, or the common type of what is known as pyorrhoea. The pain from this condition is rarely severe and therefore the patient may be unaware of it for quite a long time; but at any stage there may be an acute exacerbation with acute gingivitis, visible ulceration, bleeding from the gums, marked foetor oris, and intense pain; this is known as ulcero-membranous gingivitis, Vincent's stomatitis or Trench mouth. In severe cases the disease may spread to the other muccous surfaces of the mouth, producing ulcers, and the patient may be quite ill with a high temperature, rapid pulse and severe malaise. Perhaps it should be stressed that this rarely arises except as a complication of existing gingivitis, and therefore treatment must include the treatment of the underlying bad oral hygiene. The patients often have some form of chronic ill-health which should also be treated appropriately.

The lesions of gingivitis and pyorrhoea are usually generalized throughout the mouth, but natural pockets around erupting teeth may give rise to a local gingivitis with infection of the pocket. This is common with eruption of the third molar (wisdom tooth). It is known as *pericoronitis* and occurs most frequently around the crown of an impacted tooth which has partially erupted but can erupt no further. Classically it causes very severe pain of the throbbing type with considerable swelling, especially of the gum over the tooth, so that mastication is very painful. The pain and swelling commonly cause a reflex trismus or limitation of opening of the mouth, which may be partial or complete. As with the other types of gingivitis it often becomes chronic and subject to acute exacerbations.

Absence of teeth is no guarantee that pain is not dental, for roots are still unfortunately left behind, teeth may remain unerupted for many years, or cysts may become infected from local or haematogenous sources, when they produce symptoms similar to those of acute or chronic dento-alveolar abscess.

### OSTEOMYELITIS

Pain caused by osteomyelitis must be mentioned to distinguish it from that caused by an alveolar abscess. Almost invariably in osteomyelitis the pain is more diffuse and usually more severe. The teeth in the region are often tender to percussion, but usually several teeth are involved, whereas with an alveolar abscess, although neighbouring teeth may seem tender, the tenderness is usually much more acute in the tooth causing the abscess. Swelling is usually much greater and more diffuse in osteomyelitis, and the systemic reaction as shown by the pulse, temperature and general condition is usually much more severe. The radiographic appearances are usually easily distinguished. The abscess shows a localized area as distinct from the more diffuse involvement of bone in osteomyelitis, but it must not be forgotten that the appearance of radiographic evidence is a comparatively late finding in both of these conditions, taking approximately 10 days to develop. Except in very young children osteomyelitis is found almost exclusively in the mandible.

### *Disease of the antrum*

In the maxilla the commonest confusion of diagnosis is caused by diseases of the antrum, which may give rise to pain related to the teeth. Because the roots of the molar teeth are close to the antrum, any inflammation in this region may involve the periodontal membrane of the teeth, resulting in tenderness on biting and percussion. Here again, usually a group of teeth is affected as distinct from the more localized pain of periodontitis or alveolar abscess caused by disease of one tooth. Each year a few cases of antral carcinoma find their way to the dental hospital for the diagnosis of "dental" pain, often only after several teeth have been removed without relief. The carcinoma has, of course, advanced during this time.

*Other local causes of pain*

Of the other diseases affecting the soft tissues of the mouth and causing pain, three deserve mention. Trauma from ill-fitting or even from comparatively well-fitting dentures is very common, and the ulcers and painful areas produced may be small but are often very painful. As would be expected, they usually heal rapidly when the dentures are left out of the mouth for a few days. The second condition is aphthous stomatitis in which very painful, recurrent, small round ulcers are found in the mouth. These persist for one or two weeks, sometimes longer, and then heal spontaneously, to be followed by other batches at fairly regular intervals. Little is known of the cause though it seems probable that some are due to a virus like that of herpes simplex. It is probably a heterogenous group, and is difficult to treat. The best results are usually obtained by attention to oral hygiene and general health.

The third condition is lichen planus; it has received considerable attention recently because it is much more common than was previously supposed. It may cause a severe burning sensation in the mouth with very little to be seen. The oral manifestations may vary considerably over a period and it is therefore often necessary to examine the patient two or three times at intervals before the lesions can be found. Though it is usually a generalized disease of skin and mucous surfaces it can occur in the mouth without any lesions being found on the body. The lesions take two main forms. Commonly they appear as white dots or streaks which are often arranged in a lace-work pattern but may simulate an early leukoplakia. Less commonly there may be superficial erosions of the mucosa which are very painful and are usually associated with some of the white patches.

*Temporo-mandibular joint*

One of the conditions associated with the teeth and causing dental pain, though comparatively remote from them, is arthrosis of the temporo-mandibular joint. The precise nature of the lesion within the joint is not clearly understood. The condition usually commences with clicking in the joint, which is at first noticed only by the patient though later it may become audible to others. This may be accompanied by some pain and limitation of movement of the mandible from the beginning, but at times it continues for some years before the symptoms are severe enough to cause the patient to seek treatment. Sooner or later either this clicking becomes annoying or there is an acute exacerbation with severe pain often associated with temporary but complete limitation of movement so that the patient seeks advice. In the acute case the pain is usually fairly localized over the temporo-mandibular joint, but in the more long standing chronic cases the pain is intermittent, of varying severity, and may be referred to the temporal and parietal regions, the jaws, or to the tongue. There may be some tinitus. When all of these signs are present, it is known as Costen's syndrome, but this is rare. Much more commonly, the pain is referred to one region only, often one of the jaws. Fortunately, the diagnosis of these cases is assisted by the clicking and the abnormal movements of the mandible which are usually present. The condition is frequently related to abnormalities of the dental occlusion which can be treated, but it can arise indirectly as a result of true dental pain causing abnormal excursions of the mandible to protect the offending teeth. Diagnosis in such cases may be difficult.

It is common knowledge that pain arising from the teeth is frequently referred. This reference is usually from one molar to another in the same or opposing jaw, but it may spread over wider areas throughout the distribution of the second and third divisions of the trigeminal nerve. In acute periodontal disease, pain is fairly readily localized by the patient, but in more chronic lesions, particularly of the pulp, pain is poorly localized, and it is often referred widely.

### DIAGNOSIS

It must be obvious from this brief account of some of the possible causes of "dental" pain and from the frequency with which true dental pain occurs that diagnosis is often difficult.

There is no rule of thumb which can be applied. Only a good history and a thorough examination can guard against the many pitfalls.

The most important point in the diagnosis is to build up from the history and the present symptoms, the natural history of the condition, which should indicate the type of pathology to be considered even if it does not immediately give a conclusive answer. After this there are the special methods of examination of the teeth used by the dental surgeon to disclose caries, pulpal, periodontal and gingival disease, and the more usual examinations for antral or bony disease and for examination of the soft tissues. An elementary point which is worth mentioning is that many small lesions of the teeth and soft tissues are missed by failing to dry the tissues before examination, so that they remain obscured by saliva with its bubbles and reflections of light.

In the more obscure cases, the diagnostic injection is often of the greatest value. By means of regional block and infiltration anaesthesia which is easily produced by any dental surgeon, the source of the pain can gradually be localized to or excluded from the jaws. This is particularly of value in dealing with pain which is thought to be of psychogenic origin. The patient is asked to note the time at which relief of pain occurs and its precise duration. Saline injections can also be used to differentiate between functional and organic pains. The results are sometimes surprising.

Nothing has been said of the pain which arises after injection or extraction in the course of dental treatment. Here the cause is usually quite obvious though one must be careful, for with the prevalence of dental disease it is not uncommon for so-called post-operative pain to be caused by a cavity in a neighbouring tooth.

Any patient with pain arising after dental treatment should always be referred to the dentist as soon as possible. He knows only too well that such things happen and should always be willing to treat these patients. He is also in the very best position to know the probable causes of the pain.

### TREATMENT

The diagnosis of "dental" pain is often very simple but it is rarely wise to undertake treatment without a thorough examination of the teeth and mouth. It is not sufficient to look in the mouth and advise total extractions because the teeth are obviously stained and dirty, for in such cases attention to oral hygiene can often produce surprisingly good results, and it is often quite impossible to discover what disease is present until the teeth have been cleaned. Usually the best course is to tell the dental surgeon of the problem and ask his advice.

The treatment of the many conditions which have been mentioned is much too big a subject to be discussed here, except to say that so few of the conditions causing dental pain need treatment with systemic antibiotics that their use is rarely justified before a diagnosis can be made. In fact, their use often makes diagnosis more difficult. Local use of antibiotics carries risks and is so rarely of value that this is best avoided altogether. When a cavity can be found which is related to the pain, oil of cloves applied on a pledget of cotton wool in this cavity will often bring considerable relief, and should this fail, the common anodynes will usually succeed temporarily until dental assistance can be obtained.

## Figures and Tables

**Figure f1:**